# Application of the Adverse Outcome Pathway Concept to *In Vitro* Nephrotoxicity Assessment: Kidney Injury due to Receptor-Mediated Endocytosis and Lysosomal Overload as a Case Study

**DOI:** 10.3389/ftox.2022.864441

**Published:** 2022-04-19

**Authors:** Sebastian Jarzina, Stefano Di Fiore, Bernhard Ellinger, Pia Reiser, Sabrina Frank, Markus Glaser, Jiaqing Wu, Femke J. Taverne, Nynke I. Kramer, Angela Mally

**Affiliations:** ^1^ Department of Toxicology, University of Würzburg, Würzburg, Germany; ^2^ Fraunhofer Institute for Molecular Biology and Applied Ecology, Division Molecular Biotechnology Aachen, Aachen, Germany; ^3^ Fraunhofer Institute for Molecular Biology and Applied Ecology, Division Translational Medicine, ScreeningPort, Hamburg, Germany; ^4^ Institute for Risk Assessment Sciences, Utrecht University, Utrecht, Netherlands; ^5^ Toxicology Division, Wageningen University, Wageningen, Netherlands; ^6^ Host-microbe Interactions, Wageningen University, Wageningen, Netherlands

**Keywords:** adverse outcome pathway (AOP), nephrotoxicity, *In vitro* toxicity testing, QIVIVE, risk assessment, key event relationship

## Abstract

Application of adverse outcome pathways (AOP) and integration of quantitative *in vitro* to *in vivo* extrapolation (QIVIVE) may support the paradigm shift in toxicity testing to move from apical endpoints in test animals to more mechanism-based *in vitro* assays. Here, we developed an AOP of proximal tubule injury linking a molecular initiating event (MIE) to a cascade of key events (KEs) leading to lysosomal overload and ultimately to cell death. This AOP was used as a case study to adopt the AOP concept for systemic toxicity testing and risk assessment based on *in vitro* data. In this AOP, nephrotoxicity is thought to result from receptor-mediated endocytosis (MIE) of the chemical stressor, disturbance of lysosomal function (KE1), and lysosomal disruption (KE2) associated with release of reactive oxygen species and cytotoxic lysosomal enzymes that induce cell death (KE3). Based on this mechanistic framework, *in vitro* readouts reflecting each KE were identified. Utilizing polymyxin antibiotics as chemical stressors for this AOP, the dose-response for each *in vitro* endpoint was recorded in proximal tubule cells from rat (NRK-52E) and human (RPTEC/TERT1) in order to (1) experimentally support the sequence of key events (KEs), to (2) establish quantitative relationships between KEs as a basis for prediction of downstream KEs based on *in vitro* data reflecting early KEs and to (3) derive suitable *in vitro* points of departure for human risk assessment. Time-resolved analysis was used to support the temporal sequence of events within this AOP. Quantitative response-response relationships between KEs established from *in vitro* data on polymyxin B were successfully used to predict *in vitro* toxicity of other polymyxin derivatives. Finally, a physiologically based kinetic (PBK) model was utilized to transform *in vitro* effect concentrations to a human equivalent dose for polymyxin B. The predicted *in vivo* effective doses were in the range of therapeutic doses known to be associated with a risk for nephrotoxicity. Taken together, these data provide proof-of-concept for the feasibility of *in vitro* based risk assessment through integration of mechanistic endpoints and reverse toxicokinetic modelling.

## Introduction

The Adverse Outcome Pathway (AOP) concept has been identified as a valuable tool supporting the transition of chemical safety evaluation from testing apical endpoints in laboratory animals to more mechanism-based *in vitro* assays. AOPs are based on the recognition that toxic effects are the result of a sequence of causally linked key events (KE), ranging from the interaction of a chemical compound with a cellular target (Molecular Initiating Event, MIE) through subsequent changes at the cell and organ level to an adverse outcome (AO) (Vinken, 2013). A KE is thereby defined as a measurable or observable biological change that is essential for the progression from an MIE to an AO (Villeneuve et al., 2014). Thus, construction of AOP networks and identification of shared KEs is widely considered a promising approach to the development of batteries of mechanism-based biomarker endpoints and *in vitro* test methods for hazard assessment and chemical risk assessment. A prime example of how mechanistic understanding may form the basis of new test guidelines is the AOP on skin sensitisation (OECD, 2014). Here, *in chemico* and *in vitro* assays covering the first 3 KE that lead to skin sensitisation, i.e. reaction of an electrophilic chemical with amino acid residues in skin proteins (KE1 or MIE), keratinocyte inflammatory response and keratinocyte gene expression changes (KE2), and activation of dendritic cells (KE3) have been adopted by the OECD as test guidelines (OECD TG 442C, 442D, 442E) that can be used to assess skin sensitisation potential of chemicals (OECD, 2018a; b; 2021). While this example demonstrates the overall feasibility of utilizing AOPs for development of *in vitro* tests fit for regulatory decision making, translation of *in vivo* endpoints relevant to repeated dose systemic toxicity into suitable *in vitro* test batteries remains challenging. Moreover, besides providing information on health hazards related to exposure to a test chemical, a prime purpose of repeated dose toxicity studies in rodents is to derive a point of departure (POD) for risk assessment. Thus, use of mechanistic *in vitro* data for regulatory decisions beyond hazard identification requires not only quantitative understanding of the relationships between the KEs and the AO to predict the *in vivo* outcome, but also integration of physiologically based kinetic (PBK) modelling to translate *in vitro* concentration-response relationships to predicted *in vivo* dose-response relationships (Paini et al., 2019). This allows for derivation of a POD or—as a first validation step to assess the confidence in the approach—comparison of the predicted *in vivo* dose-response to experimentally or clinically determined *in vivo* dose-response relationships.

**FIGURE 1:**
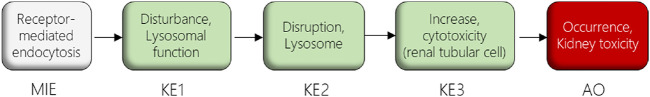
AOP receptor-mediated endocytosis and lysosomal overload. The AOP describes the sequence of key events leading to kidney injury as an AO initiated by receptor-mediated endocytosis (MIE), subsequent disturbance of lysosomal function (KE1), disruption of lysosomes (KE2) and proximal tubule cell toxicity (KE3).

In this study, we used the AOP of proximal tubule injury initiated by (receptor-mediated) endocytosis and lysosomal overload (Mally and Jarzina, 2022) as a case study to adopt the AOP concept for systemic toxicity testing and risk assessment based on *in vitro* data. In this AOP, nephrotoxicity is thought to result from receptor-mediated endocytosis (MIE) of the chemical stressor, disturbance of lysosomal function (KE1), and lysosomal disruption (KE2) associated with release of reactive oxygen species and cytotoxic lysosomal enzymes that induce cell death (KE3) (Figure 1). The AOP is based on the current understanding of the mechanism of nephrotoxicity of aminoglycoside and polymyxin antibiotics, but is also relevant to other polybasic drugs and proteins that can pass through the glomerular filter due to their low molecular weight and thus reach the proximal tubule from the luminal side. These compounds act as ligands for the cubilin:megalin-complex expressed at the apical membrane of proximal tubule cells, leading to uptake into the proximal tubule cells via receptor-mediated endocytosis (Nielsen et al., 2016). Accumulation of compounds within lysosomes leads to disruption of lysosomal function (KE1), lysosomal swelling and lysosomal membrane permeabilization (KE2) associated with release of reactive oxygen species and lysosomal proteases such as cathepsins into the cytosol (KE2), ultimately leading to cytotoxicity of proximal tubule cells (KE3).

In the present study, the described sequences of KEs served as a basis to identify *in vitro* endpoints reflecting the KEs within this AOP. Utilizing polymyxin antibiotics as chemical stressors, the dose-response for each *in vitro* endpoint was recorded in proximal tubule cells from rat (NRK-52E) and human (RPTEC/TERT1) in order to (1) experimentally support the sequence of KEs, to (2) establish quantitative relationships between KEs as a basis for prediction of downstream KEs based on *in vitro* data reflecting early KEs and to (3) derive suitable *in vitro* PODs for human risk assessment.

## Material & Methods

### Chemicals and Reagents

Unless otherwise indicated, all chemicals and reagents were purchased from Sigma-Aldrich (Taufkirchen, Germany).

### Cell Culture

The human proximal tubule cell line RPTEC/TERT1 was purchased from Evercyte Laboratories (Vienna, Austria) (Wieser et al., 2008). Cells were maintained in 75 cm^2^ cell culture flasks (Greiner, CellStar, Cat#: 658175) at 37°C and 5% CO_2_ with 12 ml medium consisting of a 1:1 mixture of DMEM no glucose (Gibco, Thermo Fischer, cat. No. 11966-025) and Ham’s F12 (Gibco, Thermo Fischer, cat. No. 21765-029) and supplemented with 10 ng/ml epidermal growth factor (EGF) (Sigma Aldrich), 10 ng/ml GlutaMAX® (Thermo Fischer), 5 μg/ml insulin, 5 μg/ml transferrin, 5 ng/ml sodium selenite, 36 ng/ml hydrocortisone, 100 U/mL penicillin and 100 μg/ml streptomycin (PAA Laboratories, cat. No. P11-010). RPTEC/TERT1 were split once a week and medium change was performed twice a week. Cells were discarded after 20–25 rounds of splitting. Unless otherwise indicated, prior to each experiment, RPTEC/TERT1 cells were cultured for 14 days in order to differentiate.

The rat kidney cell line NRK-52E was obtained from the European Collection of Authenticated Cell Cultures (ECACC) and was cultured in 75 cm^2^ cell culture flasks (Greiner, CellStar, Cat#: 658175) at 37°C and 5% CO_2_ with 12 ml DMEM containing 4.5 g/L glucose and 1.5 g/L sodium bicarbonate (Pan-biotech, cat. No. P04-03500) supplemented with 5% fetal bovine serum (Merck Millipore, cat. No. S0625, Lot No. 0865C), 10 ng/ml GlutaMAX®, 1% non-essential amino acids (Gibco, Thermo Fischer, cat. No. 11140035), 100 U/mL penicillin and 100 μg/ml streptomycin (PAA Laboratories, cat. No. P11-010). For maintenance, cells were split twice a week and were discarded after 20–25 rounds of splitting.

### TaqMan Polymerase Chain Reaction

Total RNA was isolated from cells and rat kidney tissue using Qiagen Rneasy® Mini kit (Qiagen) according to the manufaturer’s protocol. The RNA concentration was measured using a NanoDrop™ and the samples were stored at −80°C for further experiments. Total RNA was converted to cDNA using the First Strand Synthesis KitTM (Thermo Scientific™) according to the manufaturer’s protocol and cDNA synthesis was performed using a Mastercycler^©^ (Eppendorf AG, Hamburg). The samples were run with a gradient of 5 min at 25°C, 1 h at 37°C and 5 min at 70°C. The obtained cDNA samples were stored at -80°C for further experiments. TaqMan™ gene expression assay was performed as described by the manufacturer (Thermo Scientific™). For PCR with TaqMan™ probes, a cDNA concentration of 10 ng was used. TaqMan™ gene expression assay primers were used for human GAPDH (probe ID: Hs03929097_g1), LRP2 (probe ID: Hs00189742_m1), CUBN (probe ID: Hs00153607_m1), and rat Gapdh (probe ID: Rn01775763_g1), Lrp2 (probe ID: Rn00578067_m1), Cubn (probe ID: Rn00584200_m1) (Thermo Scientific™). To determine mRNA expression, quantitative real-time RT-PCR was performed using the Real-Time PCR system LightCycler® 480 (Roche) and LightCycler® Multiwell Plates 96. Cycling conditions for the LightCycler® were uracil-N-glycosylase activation for 2 min at 50°C, polymerase activation 20 s at 95°C, PCR (44 cycles) denaturation at 95°C for 3 s and annealing/extension at 60°C for 30 s.

### Western Blot Analysis

For protein extraction 1 × 10^6^ cells respectively 5 mg of a rat kidney were lysed with 300 µL freshly prepared Ripa buffer (Tris HCl (50 mM), NaCl (150 mM), Nonidet P40 (1%), Na-desoxycholat (0.25%), EDTA (1 mM), NaF (100 mM), Na_3_VO_4_ (200 mM), Protease Inhibitor Cocktail (1:50) (Thermo Fisher Scientific™). Cells were incubated with Ripa buffer for 15 min and rat kidney for 2 h at 4°C on an orbital shaker. After incubation cell lysate were centrifuged for 15 min at 10,000 rpm and 4°C and tissue samples were centrifuged for 20 min at 20,000 rpm and 4°C and then put on ice. The supernatant was then transferred to a fresh Eppendorf tube followed by protein determination using the DC assay (BioRad®). Samples and standard were then transferred into UV/VIS cuvettes and the measurement was performed at 750 nm on a spectrometer (Buck Scientific M-500) in duplicates. Protein samples were mixed with Laemmli buffer (6 x) and heated for 10 min at 100°C in a heating block. The samples were then separated on a gradient gel (Nippon Genetics Europe, FastGene® PAGE 4–12%, 8 × 10 cm) and the gel was run in MOPS buffer (Tris-base 6.06 g, MOPS 10.47 g, EDTA 0.3 g, SDS 1 g, 1,000 ml H_2_O) at 4°C for 2 h and 80 V (Hoefer Scientific Instruments SE250). Proteins were transferred to a PVDF membrane using the wet transfer method (3 g TRIS HCl, 14.4 g glycine, 800 ml H_2_O, 200 ml methanol) for 1 h at 100 V and 4°C. The membrane was shaken for 1 h in 5% milk in TBST (Tris-buffered saline with 0.05% Tween20) at room temperature to block non-specific binding sites. The primary antibody anti-megalin mouse monoclonal IgG_1_ antibody (Santa Cruz, sc-515772)) was diluted in 5% milk in TBST (1:1,000) and incubated with the membrane overnight at 4°C. The membrane was washed 3 times each 15 min with TBST on an orbital shaker. The secondary antibody (anti-mouse IgG HRP-linked antibody; Cell Signaling Technology) was diluted in 5% in TBST (1:2,500) and incubated with the membrane for 1 h at room temperature. The BioRad® Clarity Western ECL substrate was mixed 1:1 (4 ml peroxide solution +4 ml luminol solution), incubated for 2 min with the PVDF membrane and stained membrane was then detected using a Gel Doc (ImageQuant^™^ LAS 4000).

### Cytotoxicity Assay

Cells were plated into 96-well tissue culture plates (10,000 cells per well) and were allowed to grow for 48 h (NRK-52E) respectively 10 d (RPTEC/TERT1) prior to 24 h treatment with model stressors. Stock solution for each compound (2000 µM) was prepared in growth medium and serial dilutions (1:1) were made before each experiment. At the end of the 24 h treatment, cell viability was measured using the CellTiter-Glo® assay (Promega). CellTiter-Glo® reagents and buffer were mixed under light-protected conditions and 100 µL CellTiter-Glo® solution was added directly to each well followed by 2 min incubation on an orbital shaker and 10 min incubation at room temperature in the dark to stabilize the luminescence signal. The suspension was mixed via pipetting and 50 µL were transferred into white 96-well plates (PerkinElmer Inc.). The luminescence signal was measured on a multiplate reader (Mithras LB 940; Berthold). Each assay was performed in three independent experiments carried out in triplicates.

### Immunocytochemistry (LAMP1/2, Cathepsin D, Megalin)

For immunocytochemistry experiments, cells were seeded into 8-well chamber slides (Ibidi) at a density of 140 cells per µL in 300 µL growth medium and were allowed to grow for 48 h (NRK-52E) or 10 d (RPTEC/TERT1) respectively. For treatment, stock solutions (1,000 µM) of compounds dissolved in growth medium were freshly prepared and serial dilutions were made before exposure of cells for 24 h. After treatment, cells were washed twice with 150 µL PBS and fixed with 150 µL 4% paraformaldehyde in 1 x PBS for 10 min at room temperature. After fixation, cells were washed 10 min with 150 µL PBS on an orbital shaker followed by permeabilization with 150 µL 0.2% Triton X-100 in 1 x PBS for 5 min at room temperature on an orbital shaker. The permeabilized cells were washed twice with 1 x PBS (150 µL) for 10 min at room temperature on an orbital shaker and then incubated with 150 µL 5% BSA dissolved in 1 x PBS for 1 h at room temperature to block unspecific binding sites. After blocking, 100 µL primary antibody diluted 1:100 in 1% BSA in 1 x PBS were directly pipetted onto the cells and incubated overnight at 4°C. The following antibodies were employed for NRK-52E cells: Anti-LAMP-1 monoclonal IgG_1_ kappa mouse antibody (Santa Cruz; sc-20011), anti-Cathepsin D mouse monoclonal IgG_2_ kappa antibody (Santa Cruz; sc-377124); for RPTEC/TERT1 cells: Anti-LAMP-2 monoclonal mouse antibody (Santa Cruz; sc-18822), anti-Cathepsin D mouse monoclonal IgG_2_b kappa (Novus Biologicals; NBP1-04278); for both cell lines anti-megalin mouse monoclonal IgG_1_ antibody (Santa Cruz, sc-515772).

After incubation with primary antibody, cells were washed three times for 15 min with 0.2% Tween in 1 x PBS (150 µL) on an orbital shaker. Cells were incubated for 1 h at room temperature with 100 µL CFL 488-conjugated mouse IgG kappa binding protein (1:50) (m-IgGκ BP-CFL 488; Santa Cruz, sc-516176). After incubation the cells were washed three times for 15 min with 0.2% Tween in 1 x PBS on an orbital shaker and once with 150 µL sterile water. Phalloidin-tetramethylrhodamine B isothiocyanate (TRITC) was used to stain the cytoskeleton and was diluted in 100% methanol to a stock concentration of 0.1 mg/ml and further diluted in 1 x PBS to a working concentration of 0.095 µM. After cells were washed once with 1 x PBS for 10 min at room temperature 150 µL phalloidin-TRITC working solution was added and the cells were incubated on an orbital shaker at room temperature for 60–90 min. After incubation, phalloidin-TRITC solution was aspirated, and the cells were washed once with 1 x PBS. Chamber slides were allowed to dry for 15 min at room temperature. To preserve fluorescence 50–100 µL DAPI mounting medium (Vectashield®; Vectorlab, Biozol) was added to the cells and chamber slides were stored at 4°C in the dark until image acquisition.

Images were taken with a TCS SP5 II confocal microscope with an HCX PL APO lambda blue 63.0 × 1.40 OIL UV objective (Leica Microsystems, Wetzlar, GER) (*n* = 10 cells/treatment group). The maximum excitation and emission settings for image acquisition were the following: Vectashield® antifade mounting medium with DAPI (λ_ex_ 360 nm & λ_em_ 460 nm), for CFL 488-conjugated mouse IgG kappa binding protein (λ_ex_ 490 nm & λ_em_ 525 nm) and phalloidin-TRITC (λ_ex_ 545 nm & λ_em_ 573 nm). Before quantification, images were blinded and randomized. Quantification of fluorescence intensity was performed using NIH Image® software. Using the tool “Plot Profile” we generated a two-dimensional graph of the intensity of pixels along a reference line (6.35 µm) drawn across the cytoplasm of cells. The mean values of the pixel intensities were automatically calculated by the software and imported in Excel for further processing.

### High Content Imaging Assays

For high content imaging, NRK-52E and RPTEC/TERT1 cells were cultivated in µ-clear half well area microtiter plates (Greiner) according to the protocol described for the cytotoxicity assay with slight modification. Briefly, NRK-52E cells were seeded at 5,000 cells in 100 µL growth medium per well and were incubated under standard growth conditions for 48 h prior to treatment. RPTEC/TERT1 cells were seeded at 15,000 cells in 100 µL growth medium per well and incubated under standard growth conditions for 7–8 days, until cells reached confluence and started to differentiate. This was indicated by the formation of three-dimensional structures referred to as domes which were identified by regular microscopic inspection of the plates. Only differentiated cell cultures with detectable domes were assayed. Cells of both cell lines were treated with serially diluted Polymyxin B sulfate (concentration range 7.8 to 2,000 µM) for 1, 2, 4, 6, or up to 24 h. The assay was carried out on three biological replicates defined as three different non-consecutive passages of the cell culture. Each biological replicate at each treatment concentration was assayed in triplicate wells per plate.

Following treatment, cells were stained with two different combinations of fluorescent dyes: NRK-52E cells with 50 pg/μL LysoView™ 633 (Biotium) and 1 µM SYTO 16 (Thermo Scientific), respectively, for lysosomes and nuclei; RPTEC/TERT1 cells with 10 nM LysoTracker™ Red (Thermo Scientific) and 10 ng/μL Hoechst 33342 (Merck-Sigma Aldrich), respectively, for lysosomes and nuclei. For each cell line a staining solution was obtained by mixing the lysosomes and nuclei dyes in cell culture medium at the concentrations indicated above. The cells were stained by replenishing the medium for 100 µL of staining solution per well followed by incubation at 37°C and 5% CO_2_ for 30 min before imaging.

Images were acquired with a fully automated fluorescence confocal Opera™ QEHS system (PerkinElmer, United Kingdom) using a ×40 air UPlanApo objective (Olympus) with 0.85 numerical aperture. NRK-52E cells stained with the dyes SYTO 16 and LysoView™633 were imaged with the laser lines 488 and 635 nm, using a 488/561/635 nm primary dichroic mirror, a 510 nm long pass (LP) detection dichroic mirror, a 540/75 nm band pass (BP) emission filter for SYTO 16 and a 690/50 nm BP emission filter for LysoView™ 633. RPTEC/TERT1 cells stained with the dyes Hoechst 33342 and LysoTracker™Red were imaged with the laser lines 405 and 561 nm, using a 405/561/635 nm primary dichroic mirror, a 580 nm LP detection dichroic mirror, a 450/50 nm BP emission filter for Hoechst 33342 and a 600/40 nm BP emission filter for LysoTracker™Red. To prevent cross talk of the fluorescent signals, each dye was imaged consecutively using the combination of lasers and filters described above. The settings for laser power, exposure time and focal height were kept constant for each cell line across all the experiments. For NRK-52E cells images were acquired at 25 randomly distributed positions in a well at a single focal plane whereas for RPTEC/TERT1 cells due to the development of dome structures images were acquired at 9 random positions at three focal planes each 1 μm apart.

The images were analyzed by custom-made image analysis scripts either with the imaging analysis software Acapella (version 2.8 PerkinElmer) or Columbus (version 2.4.0 PerkinElmer). The scripts included modules for the identification and counting of the cell nuclei to extrapolate the number of cells per well, for definition of a cytoplasm surrounding the nucleus, for identification and counting of the lysosome vesicles within the cytoplasm. Additional cellular parameters included the nuclei size, intensity, the cell size (cell area), the lysosome size (lysosome area), intensity and the number of lysosomes normalized by the cell area to compensate for difference in size of the cells. For RPTEC/TERT1 cells an additional script module was included to achieve maximum projection of the three optical planes onto a two-dimensional image. These images were then analyzed as described above. The image analysis results were imported as. csv (comma separated values) or ascii files into Excel 2013 or OriginPro for further data analysis.

### LC-MS/MS Analysis of Intracellular Polymyxin B and Colistin

NRK-52E and RPTEC/TERT1 cells were seeded in 12-well plates (120,000 cells per well) and were allowed to grow for 48 h (NRK-52E) or 10 d (RPTEC/TERT1) until they reached 100% confluence. After cells achieved 100% confluence, the supernatant was aspirated, and the cells were washed twice with 1 x PBS. Cells were treated with polymyxins at a final concentration of 34 µM for 24 h. Samples were taken at five different treatment time points (1–2 min, 1, 3, 6, and 24 h). In addition, a recovery sample was taken 24 h after the end of the 24 h treatment period. To this end, the supernatant was aspirated after 24 h treatment and the cells were washed three times with 1 x PBS to remove polymyxin B or colistin residues. Fresh growth medium was added, and the cells were incubated for another 24 h. At the end of each experiment, the cells were washed 3 times with 1 x PBS and trypsinized by adding 200 µL of trypsin. Detached cells were washed with 1,000 µL 1 x PBS and the cells were transferred to LoBind™ tubes (Eppendorf). Cell count was determined using a Fuchs-Rosenthal counting chamber (Hartenstein). Cells were centrifuged for 5 min at 4°C at 10.000 rpm and the supernatant was aspirated. Cells were resuspended in 1,000 µL 1 x PBS and centrifuged for 5 min at 4°C at 10.000 rpm. After centrifugation, the supernatant was aspirated, and the cell pellet was resuspended in 250 µL ammonium acetate buffer (10 mM) and 250 µL of 30% methanol with 1% acetic acid containing polymyxin B respectively colistin as an internal standard. Cells were sonicated for 10 min and centrifuged for 5 min at 4°C at 14.000 rpm. The supernatant was transferred quantitatively into new LoBind™ tubes (Eppendorf) and dried in a centrifugal vacuum concentrator for 2–3 h. To measure compound plastic binding, the empty 12 well plates were washed with 1.5 ml acetonitrile on an orbital shaker for 2 h and supernatant was collected. For sample purification via solid phase extraction, SPE cartridges Strata-X 33 µm polymeric reversed phase, 10 mg/1 ml (Phenomenex) were used. In the conditioning step the cartridges were rinsed with 200 µL methanol (100%) and subsequently with 200 µL ddH_2_O to activate the sorbent bed. The dried samples were dissolved in 200 µL 1% acetic acid and transferred onto the SPE cartridge. The SPE cartridge were rinsed once with 200 µL 1% acetic acid. Impurities were removed by rinsing the cartridge once with 200 µL 10% methanol. A fresh LoBind™ tube (Eppendorf) was placed under the SPE cartridge to eluate the samples by rinsing twice with 200 µL 90% methanol containing 1% acetic acid. The samples were dried in a centrifugal vacuum concentrator for 2–3 h. All samples were stored at −20°C until LC-MS/MS analysis.

A triple quadrupole mass spectrometer equipped with a Turbo-Ion® Spray source (QTrap API 2000; AB Sciex Instruments) was used for analyses. The mass spectrometer was coupled to an Agilent 1100 HPLC system consisting of a binary pump system, a vacuum degasser and an autosampler (Agilent 1,100). Chromatographic separation was performed at 40°C with a column oven (Knauer) on a Synergi Hydro-RP column (2 mm × 150 mm, 4 μm, 80 Å; Phenomenex) coupled to a precolumn (SecurityGuard™ Cartridges, AQ C18 4 × 2.0 mm) using a binary step gradient at a flow rate of 0.2 ml/min with the following gradient: 0 min 100% A, 15 min 0% A, 20 min 0% A, 21 min 100% A, and 30 min 100% A. Mobile phase consisted of 3% acetonitrile containing 1% acetic acid and (solvent A) and 97% acetonitrile containing 1% acetic acid (solvent B). The injection volume was set to 10 µL and the total analysis time was 30 min. Following chromatographic separation, multiple ion monitoring was used to detect the triply charged, twice charged, and single charged ions of polymyxins (Table 1). Limit of detection (LOD) and limit of quantification (LOQ) was calculated by using the signal-to-noise ratio (S/N) method, where a signal-to-noise ratio of three was used to estimate the LOD and a signal-to-noise ratio of ten was used to estimate the LOQ.

**TABLE 1:** LC-MS/MS parameters for MRM detection of polymyxin B1, polymyxin B2, colistin A, and colistin B, including mass transitions for the triple [M+3H]^3+^, double [M+2H]^2+^and single [M+H]^+^ charge ions; declustering potential (DP), entrance potential (EP), collision entrance potential (CEP), collision energy (CE), cell exit potential (CXP), and retention time (RT).

Charge	Analyte	Transition [m/z]	**DP [V]**	**EP [V]**	**CEP [V]**	**CE [V]**	**CXP [V]**	**RT [min]**
**[M+3H]** ^**3+**^	Colistin A	386 → 101.1	31	7.5	16.21	27	2	11.2
	Colistin B	390.7 → 101.1	31	8	16.33	27	2	11.0
	Polymyxin B2	397.3→ 101.1	51	7	16.49	29	2	11.1
	Polymyxin B1	402.1→ 101.1	31	8	16.61	27	2	11.4
**[M+2H]** ^**2+**^	Colistin A	578.6 → 101.1	66	9	21.06	53	2	11.2
	Colistin B	585.6 → 101.1	66	9.5	21.24	49	2	11.0
	Polymyxin B2	595.6 → 101.1	66	12	21.49	49	2	11.1
	Polymyxin B1	602.6 → 101.1	71	9.5	21.66	47	2	11.4
**[M+H]** ^**+**^	Colistin A	1,156.03 → 302.2	151	11	35.6	77	4	11.2
	Colistin B	1,170.03 → 302.2	151	11	35.95	75	4	11.0
	Polymyxin B2	1,190.05 → 302.3	151	12	36.46	79	4	11.1
	Polymyxin B1	1,204.04 → 302.2	151	11	36.81	79	4	11.4

To determine the cell volume of NRK-52E and RPTEC/TERT1 cells, a cell culture flask with 70–80% confluent cell layer was trypsinized until cells were rounded. Images were taken from rounded cells on an ECLIPSE 55i microscope (Nikon) (*n* = 28) and the diameter of individual cells was measured using NIH ImageJ® software. Single cell volume was calculated based on the method described by Zhang and colleagues for the volume of a sphere where *d* is the diameter of a single cell is (V_Sphere_ = (1/6)*π*d³) (Zhang et al., 2015). The volume for each cell was calculated based on this equation and mean values of cell volume were used to calculate total volume of viable cells in the cell samples (V_Total cell volume_ = n_Cell number_ * V_Mean cell_). Intracellular compound concentration was finally calculated by the total amount of drugs measured in samples divided by the total volume of viable cells in the samples (c_Compound intracellular_ = c_Compound in sample_/V_Total cell volume_).

### Determination of Aprotinin Uptake to Assess Endocytic Activity

Cells were seeded at a density of 140 cells per µL in 300 µL growth medium per chamber on 8-well chamber slides (Ibidi®) and were allowed to grow for 48 h (NRK-52E) or 10 d (RPTEC/TERT1) respectively. A 100 μg/ml Alexa Fluor 488 labelled aprotinin solution was added to the cells (300 µL) and cells were incubated for 4 h at 37°C and 5% CO_2_ atmosphere. After incubation, the aprotinin solution was aspirated and cells were washed 3 times with 1 x PBS on an orbital shaker followed by fixation and DAPI staining. Images of labelled cells were acquired with a TCS SP5 II confocal microscope with an HCX PL APO lambda blue 63.0 × 1.40 OIL UV objective (Leica Microsystems) (*n* = 4 images per group). Images were quantified with the ImageJ® software using the tool “Plot Profile” to measure Alexa-488 intensity along a reference line drawn across the cytoplasm of control and exposed cells. Fluorescence intensity of Alexa-488 labelled aprotinin was divided by the number of nuclei per image and results were plotted as mean fluorescence intensity per nucleus.

### Response-Response Analysis for Prediction of Downstream Key Events

Response-response plots generated from polymyxin B data were used to establish the quantitative relationships between KE (KER) and these plots and associated mathematical equations were then used to predict the cytotoxicity of colistin and polymyxin B nonapeptide based on experimental data reflecting KE1. Response-response analysis involved calculation of additional data points and curve fitting for each KE using PROAST webtool, generation of response-response plots for polymyxin KE data, curve fitting and determination of regression equations using PROAST webtool and GraphPad Prism to describe the KER between KE1 and KE2 and between KE2 and KE3. As a proof-of-concept, these plots and associated mathematical equations were then used to predict cytotoxicity (KE3) as a late KE based on the response of an upstream KE to other putative stressors of this AOP, i.e., colistin, polymyxin B nonapeptide, and CdCl_2_. This process involved generation of experimental KE data for each stressor, i.e., KE1 and the KE to be predicted, and subsequent calculation of additional data points and curve fitting for KE1 data using PROAST webtool. Based on the mathematical description of the generated KER, the dose-response relationship between test compound concentration and downstream KEs was predicted, i.e. the mathematical description of the relationship between KE1 and KE2 generated using experimental polymyxin data was used to predict the dose-response relationship between the stressor concentration and KE2 disruption of lysosomes, while the response-response relationship between KE2 and KE3 cytotoxicity was subsequently used to derive the predicted dose-response for cytotoxicity (KE3) for each stressor. In the final step, curve fitting and regression analysis were performed using PROAST webtool and GraphPad Prism software, and the measured and predicted cytotoxicity of the test compound were compared.

### QIVIVE

**FIGURE 2:**
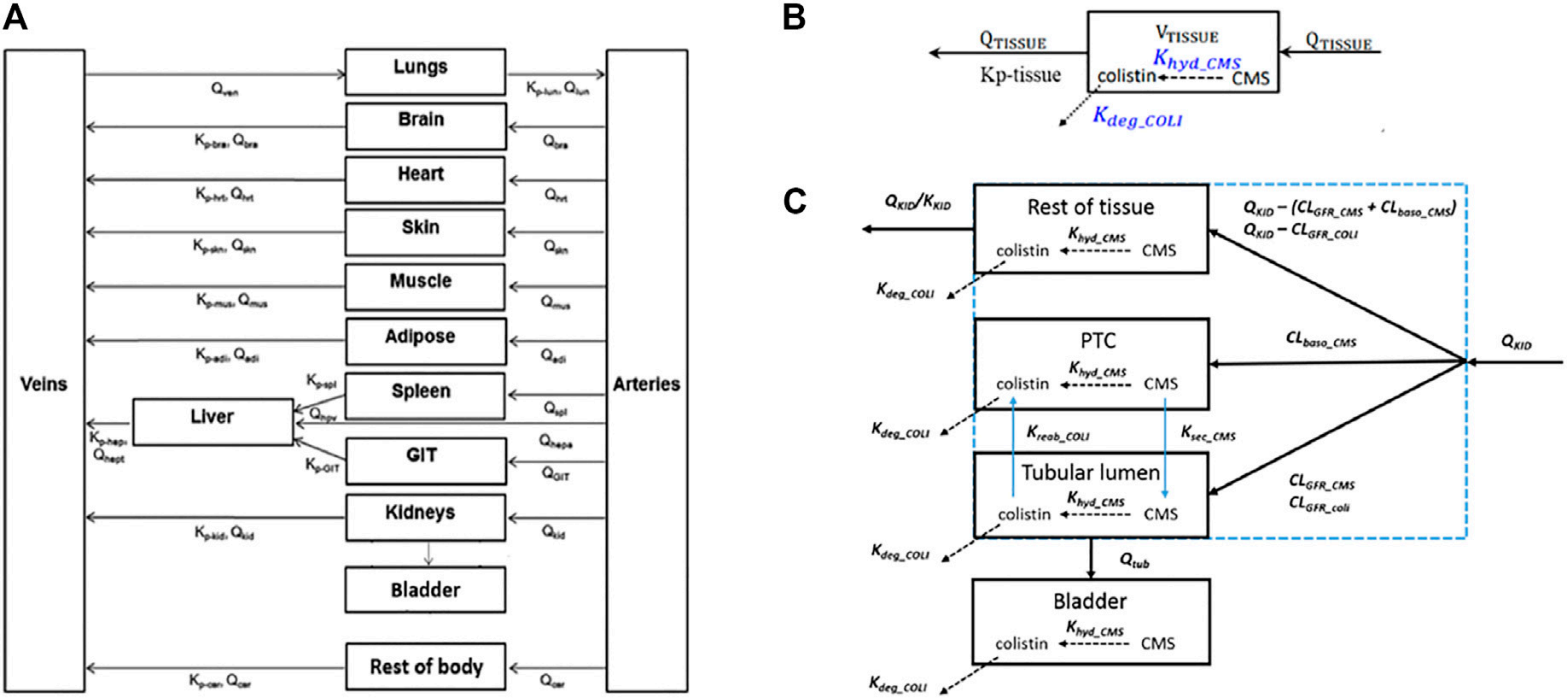
Schematic representation of the model **(A)** and the distribution mechanisms of compounds in the kidneys **(C)** or to the other tissue compartments **(B)**. Figure **(A)** was adapted from Bouchene et al. (2018). Figure **(B,C)** were adapted from Viel et al. (2018). The terms refer to: V_TISSUE_, tissue volume; Q_TISSUE_, tissue blood flow; Kp-tissue, partition coefficient; K_KID_, partition coefficient for kidneys; Q_KID_, kidney blood flow; Q_TUB_, tubular fluid flow; CL_GFR_CMS_ and CL_GFR_COLI_, glomerular filtration clearance of CMS and colistin. CL_baso_CMS_, clearance of CMS from the blood to PTC across the basolateral membrane of PTC; K_reab_coli_, reabsorption rate of colistin from the tubular lumen to PTC across the apical membrane of PTC; K_sec_CMS_, secretion rate of CMS from PTC to the tubular lumen across the apical membrane of PTC.

A generic PBPK model was used to simulate concentrations of polymyxin antibiotics in rat and human tissue, including the proximal tubule cells (PTC). Using the models of (Viel et al., 2018) and (Bouchene et al., 2018) as starting points, the model was constructed with twelve tissue compartments, representing lungs, brain, heart, skin, muscles, adipose, liver, spleen, gastrointestinal tract (GIT), kidneys, bladder and the rest of body, connected by blood circulation. The kidney compartment is divided into three sub-compartments, representing PTC, tubular lumen, and the rest of tissue. A schematic representation of the model and the distribution mechanisms of compounds to the tissue compartments are shown in Figure 2. The model was parameterized using system-specific values from) (Brown et al., 1997) and chemical-specific values from (Bouchene et al., 2018). The model was evaluated by comparing the simulated values to the *in vivo* observed data of plasma or kidney concentrations of colistin or its prodrug colistimethate sodium (CMS) following single or repeated intravenous administration of colistin or CMS (Li et al., 2004; Marchand et al., 2010; Yousef et al., 2011; Lin et al., 2017; Sivanesan et al., 2017). Model simulations of plasma and tissue concentrations in rats and humans were within 2 to 5-fold of available measured concentrations. The model was subsequently used to predict dose-dependent rat and human *in vivo* kidney injury using PBK modeling-facilitated reverse dosimetry of the *in vitro* concentration-effect relationship for polymyxin B in the CellTiter-Glo® assay with RPTEC/TERT1 (downstream KE) collected in this study, thus providing a proof-of-principle that acute kidney injury in humans can be predicted for both rats and humans without the need for *in vivo* studies (Omwenga et al., 2021; Punt et al., 2021). Predictions on dose-dependent kidney injury inhibition in humans were evaluated by comparison with available clinical data (Falagas and Kasiakou, 2006).

## Results

### Identification of Suitable *In Vitro* Endpoints to Assess the Key Events

Expert knowledge and publicly available literature were used to identify suitable *in vitro* endpoints for the KEs. To this end, the Comparative Toxicogenomic Database (CTD) (http://ctdbase.org/), a publicly accessible data repository which contains curated information about chemical-gene/protein interactions was queried to retrieve data on putative chemical stressors for the AOP.

CTD database search retrieved only few chemical-gene interactions for polymyxins as chemical stressors of the AOP (polymyxin B—40 gene data; colistin—15 gene data; polymyxin B nonapeptide—0 gene data), which included mostly genes associated with drug metabolism and cell death. In contrast, larger datasets were available for gentamicin and cadmium chloride (gentamicin—2,402 gene data; cadmium chloride—6,086 gene data). Analysis of data related to these two compounds revealed up-regulation of lysosome-associated membrane protein (LAMP) genes as a common response linked to the lysosomal pathway, kidney disease and acute kidney. LAMP-1/2 present the most abundant proteins of the lysosomal membrane and are thought to play a key role in maintaining the functional and structural integrity of lysosomes. Lysosomal storage disorders are characterized by disturbed LAMP expression (Meikle et al., 1997; Hua et al., 1998; Kroemer and Jäättelä, 2005; Ginet et al., 2009; Appelqvist et al., 2011; Damaghi et al., 2015). Thus, LAMP-1/2 expression was considered a potential *in vitro* endpoint for KE1 (*Disturbance of lysosomal function*). Besides LAMP-1/2, high content imaging using lysosomal dyes such as LysoView™ and LysoTracker™ was identified as an *in vitro* endpoints reflecting disturbed lysosomal function (KE1) and/or lysosomal disruption (KE2). LysoView™ and LysoTracker™ are fluorescent dyes that accumulate within lysosomes due to the low pH in these organelles, and can thus be used for imaging lysosome localization and morphology in fixed and live cells.

Further analyses that included vancomycin as an additional potential stressor revealed a common match for genes encoding cathepsin A, cathepsin C, cathepsin D and cathepsin S and an association of cathepsins with the lysosomal pathway, kidney disease and acute kidney injury. Cathepsins are lysosomal proteases that play a vital role in intracellular protein degradation and energy metabolism. Lysosomal membrane permeabilization (LMP) and rupture of the lysosomal membrane result in release of intralysosomal components into the cytoplasm, and has been identified as a key step in cell death signaling (Johansson et al., 2010; Quiros et al., 2010). In particular, the aspartyl protease cathepsin D has been associated with apoptosis (Mrschtik and Ryan, 2015), and is expressed at medium to high abundance in proximal tubule cells, including RPTEC/TERT1 cells (Wilmes et al., 2013). Release of cathepsins into the cytoplasm has been reported in response to treatment with aminoglycosides (Quiros et al., 2011), which are putative stressors for this AOP. Thus, analysis of the release of cathepsin D was considered as a potential *in vitro* endpoint reflecting KE2 (*Disruption of lysosomes*).

As rupture of lysosomes and associated release of cathepsins ultimately leads to proximal tubule cell death cells (KE3), cytotoxicity determined by the CellTiter-Glo® assay and by high content imaging of cell nuclei stained with Hoechst 33342 (RPTEC/TERT1) or SYTO 16 (NRK-52E) were selected as *in vitro* read-outs reflecting KE3.

### High Content Imaging Assays

**FIGURE 3:**
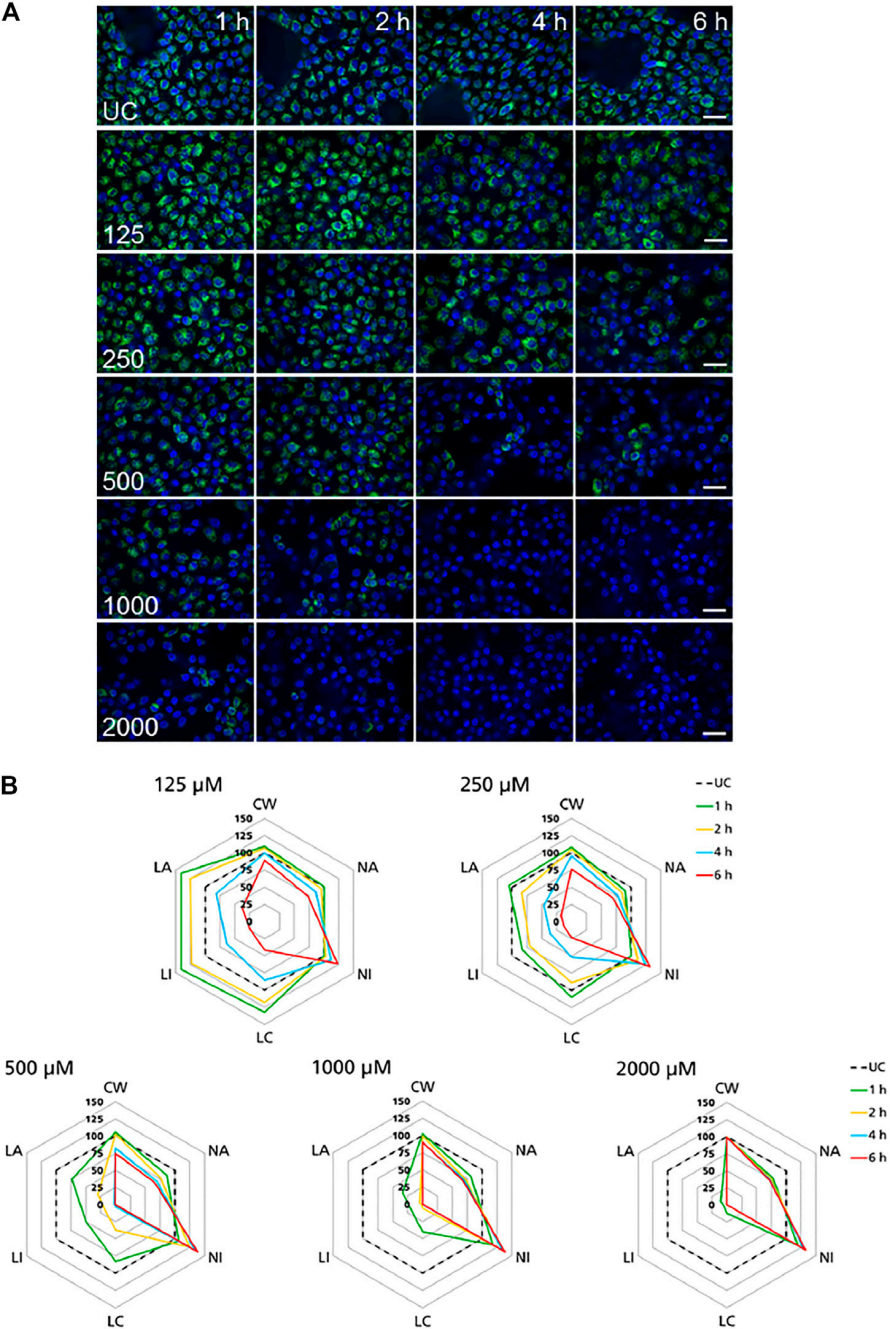
Time-resolved analysis of the effect of polymyxin B on RPTEC/TERT1 cells. **(A)** Representative confocal microscopy images of cells treated with polymyxin B (only selected concentrations are shown). Each row shows a different concentration (µM), each column represents a treatment time. Lysosomes stained with the fluorescent dye LysoTracker™ Red are shown in green. Nuclei stained with the fluorescent dye Hoechst 33342 are shown in blue. Scale bar = 20 μm. **(B)** Radar or spider-web diagrams based on relative values for selected image analysis parameters normalized against untreated control cells (UC) set to 100% (black dashed line). Each coloured line represents a time point between 1 and 6 h treatment. Each diagram refers to a selected concentration (only concentrations between 125 and 2000 µM are shown). UC = untreated control cells; CW = cells per well; NA = nuclei area; NI = nuclei intensity; LC = lysosomes per cell area; LI = lysosome integrated intensity; LA = total lysosome area.

**FIGURE 4:**
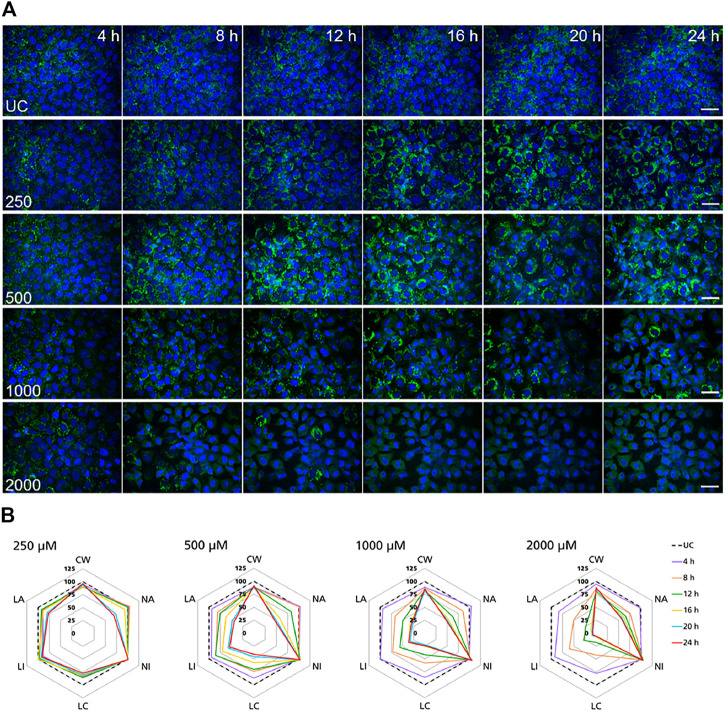
Time-resolved analysis of the effect of polymyxin B on NRK-52E cells. **(A)** Representative confocal microscopy images of cells treated with polymyxin B (only selected concentrations are shown). Each row shows a different concentration (µM), each column represents a treatment time. Lysosomes stained with the fluorescent dye LysoView™ 633 are shown in green. Nuclei stained with the fluorescent dye SYTO16 are shown in blue. Scale bar = 20 μm. **(B)** Radar or spider-web diagrams based on relative values for selected image analysis parameters normalized against untreated control cells (UC) set to 100% (black dashed line). Each coloured line represent a time point between 4 and 24 h treatment. Each diagram refers to a selected concentration (only concentrations between 250 and 2000 µM are shown). UC = untreated control cells; CW = cells per well; NA = nuclei area; NI = nuclei intensity; LC = lysosomes per cell area; LI = lysosome integrated intensity; LA = total lysosome area.

High-content imaging was applied as a preliminary robust assay to evaluate the time-scale of the occurrence of selected KEs and to determine the specificity of lysosomal KEs through screening additional nephrotoxins that are not considered to operate via this AOP. To support the temporal sequence of events, the effect of the model stressor polymyxin B was measured by time-resolved analysis in RPTEC/TERT1 and NRK-52E cells treated for 1–6 h or up to 24 h, respectively. Automated confocal fluorescence microscopy of cells stained with fluorescent dyes for lysosomes and for the cell nucleus followed by image analysis allowed for the simultaneous evaluation of multiple cellular parameters describing lysosome function and morphology as well as cytotoxicity (Figures 3A, 4A). Representative images of treated RPTEC/TERT1 (Figure 3A) and NRK-52E cells (Figure 4A) clearly showed that lysosomal disturbance culminated in lysosomal membrane rupture as indicated by a consistent decrease in the number of lysosomes and diffusion of the fluorescent lysosomal dyes in the cytoplasm. This event anticipated the onset of cytotoxicity as shown by nuclei shrinkage and increase in intensity of the nuclei staining likely due to chromatin condensation (Figures 3A, 4A). This was especially visible at high concentrations after extended time of treatment. The effect of polymyxin B was quantified by image and data analysis using spider web diagrams (Figures 3B, 4B) based on six selected representative parameters: three reporting on cytotoxicity such as number of cells per well (CW), nuclei area (NA) and nuclei intensity (NI) and three lysosomal descriptors including median of the number of lysosomes per cell area (LC) obtained by the number of detected lysosomes in each cell normalized by the cell size or cell area, lysosomal size in pixel as total lysosomal area per cell (LA), lysosome intensity (LI) as mean of the median integrated pixel intensity of each selected lysosome per cell (the integrated intensity took into account the size or area of the lysosome). The selected parameters were normalized at each time point against the value of the corresponding parameter in untreated control cells (UC), which was set to 100%. In RPTEC/TERT1 cells, lysosomal effects were already measurable after 1 and 2 h treatment at 125 µM and after 1 h at 250 μM, whereas nuclear changes were first recorded after 4 and 6 h treatment at 125 µM or after 2 htreatment at 250 μM, respectively. Decrease in nuclei area coupled with an increase in nuclei intensity as compared to untreated control cells was first detectable after 4 h treatment at 125 µM and was associated with a decrease in lysosomes per cell area, total lysosomal area per cell and lysosome intensity due to lysosome rupture. This was even more evident after 2 h treatment at 250 µM. Interestingly, lysosomal parameters firstly increased and then started to drop at 125 μM, likely reflecting lysosomal swelling due to disturbed lysosomal function in response to compound accumulation. At higher concentrations, both lysosomal and cytotoxicity parameters were already strongly altered in a concentration dependent manner after 1 or 2 h treatment (Figure 3B).

In NRK-52E cells, a similar effect was clearly recorded in cells treated at 1,000 and 2000 µM (Figure 4B). Compared to untreated cells changes in lysosomal parameters measured after 4 h treatment were not associated with significant changes in nuclei area and intensity, whereas starting at 8 h more pronounced effects on lysosomal endpoints were already accompanied by nuclear changes. At 500 μM, similar effects first appeared at 12 h whereas at 250 µM significant changes in nuclei area and marginal changes in nuclei intensity appeared at 20 h. Overall, these data confirmed the time and dose-dependent effects of polymyxin B and indicated that NRK-52E cells were in general less susceptible to polymyxin toxicity than RPTEC/TERT1 cells.

The high content imaging data showed that for two lineages of proximal tubule cells, disturbance of lysosome function preceded the onset of cytotoxic effects for polymyxin B as an exemplary stressor for this AOP. Moreover, using high content screening of additional nephrotoxins, including presumed additional stressors for this AOP (i.e., colistin, polymyxin B nonapeptide, vancomycin, gentamicin, CdCl_2_) as well as compounds that are thought to cause kidney toxicity via inhibition of mitochondrial DNA polymerase γ (cidofovir, tenofovir, tenofovir disoproxil fumarate) or covalent protein binding (i.e., S-(1,2,2-trichlorovinyl)-L-cysteine (TCVC), acetaminophen and 4-aminophenol) (Birk et al., 2021; Mally and Jarzina, 2022), the most prominent effects on lysosomal endpoints were induced by stressors of this AOP, even though minor changes were also evident in response to compounds that act primarily via mechanisms unrelated to lysosomes, particularly at concentrations at which cytotoxicity occurred (Supplementary Figure S1).

### *In Vitro* Key Event Data Obtained From Model Chemical Stressors

To further experimentally support the developed AOPs and to establish the quantitative relationships between KEs, proximal tubule cells from rat (NRK-52E) and human (RPTEC/TERT1) were treated with model compounds and selected *in vitro* endpoints were assayed for each KE.

**FIGURE 5:**
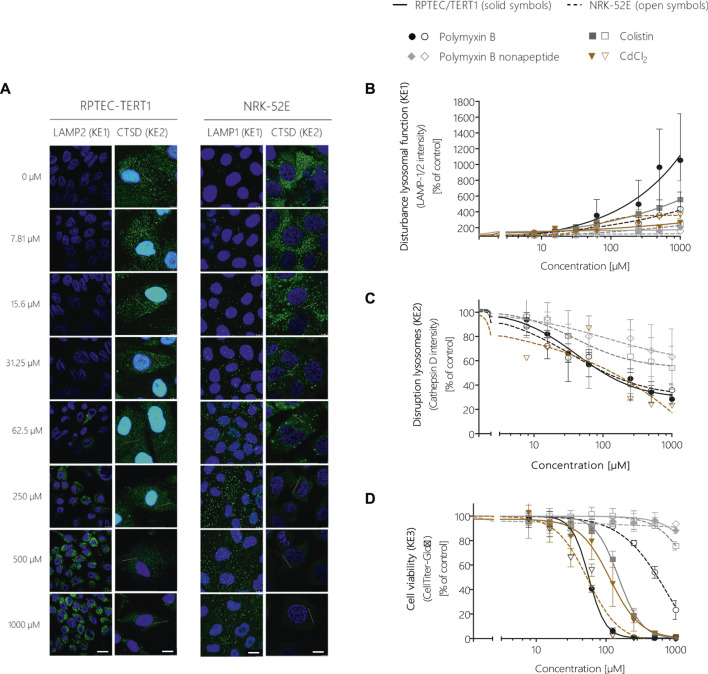
Quantitative analysis of the effect of polymyxin B across the KEs of the AOP receptor-mediated endocytosis and lysosomal overload. Confocal immunofluorescence images of LAMP-1/2 (green; scale bar: 15 µm) and cathepsin D (green, scale bar: 10 µm) in RPTEC/TERT1 and NRK-52E cells treated with polymyxin B for 24 h **(A)**. Cell nuclei were stained with DAPI (blue). Untreated kidney tubule cells showed poor LAMP-1/2 staining, while treatment with polymyxin B resulted in a concentration-dependent increase in LAMP-1/2 **(A,B)**. Cathepsin D staining in untreated cells appeared with characteristic punctual structures throughout the cytosol, consistent with its lysosomal localization. Treatment with polymyxin B caused a concentration-dependent re-distribution of the punctate cathepsin D staining to a more diffuse staining throughout the cytosol, indicative for lysosomal membrane permeabilization with release of cathepsin D **(A,C)**. Concentration-response curves obtained from LAMP-1/2 (KE1) and cathepsin D (KE1) immunofluorescence analysis **(B,C)** and cell viability (KE3) **(D)** following treatment of RPTEC/TERT1 (solid lines, solid symbols) and NRK-52E cells (dashed lines, open symbols) with polymyxin B, colistin, polymyxin B nonapeptide and CdCl_2_ as putative stressors for this AOP. Note that technical issues precluded generation of cathepsin D data on colistin, PBNP, and CdCl_2_ in RPTEC/TERT1 cells **(C)**. All experiments were performed in three independent experiments carried out in triplicates. Data are presented as mean ± SD fold change (*n* = 3).

Image analysis of LAMP-1/2 immunofluorescence in RPTEC/TERT1 and NRK-52E cells treated with polymyxin B for 24 h revealed a significant, concentration-dependent increase in LAMP-1/2 expression in response to polymyxin B (Figures 5A,B), reflecting changes in lysosomal function and/or structure (KE1). As a potential *in vitro* endpoint for KE2, release of the lysosomal enzyme cathepsin D into the cytoplasm was assayed by immunocytochemistry. In untreated cells, intralysosomal localization of Cathepsin D was evident by an intense punctual staining throughout the cytoplasm (Figure 5A). After 24 h treatment of RPTEC/TERT1 and NRK-52E cells with polymyxin B, cathepsin D staining became more diffuse throughout the cytoplasm (Figure 5A). Quantitative image analysis revealed a concentration-dependent decrease in fluorescence intensity (Figure 5C), indicating redistribution of cathepsin D from lysosomes into the cytoplasm. A concentration-dependent decrease in cell viability was observed in both cell models after 24 h treatment with polymyxin B, whereby RPTEC/TERT1 cells were found to be significantly more sensitive to polymyxin B cytotoxicity as compared to NRK-52E cells as already shown by high content imaging data. The increased sensitivity of RPTEC/TERT1 cells to polymyxin B was also evident by LAMP-1/2 analysis (Figure 5B). Essentially the same effects were observed in cells treated with the structural analogues of polymyxin B, i.e., colistin and polymyxin B nonapeptide, whereby the rank order of effects for the 3 polymyxin derivatives was consistent across all KEs, with polymyxin B being most active, and polymyxin B nonapeptide being the least toxic. In contrast to the polymyxin derivatives, cadmium chloride (CdCl_2_) was found to be more toxic in NRK-52E cells as compared to RPETC-TERT1 cells. Importantly, Cd had only minor effects on LAMP1/2 expression (KE1) despite significant cytotoxicity (KE3) (Figures 5B,D).

In addition to the integrated approach for prediction of *in vivo* nephrotoxicity described in 3.6, *in vitro* effect concentrations of polymyxins were compared to plasma concentrations at relevant exposures to obtain a first rough estimate of risk. From our KE dose-response data, BMC_10_s were calculated as *in vitro* points of departure (POD) and the margin-of-exposure (MOE) approach was applied to human and rat plasma concentrations achieved after therapeutic and/or nephrotoxic doses. The *in vitro* PODs obtained in RPTEC/TERT1 cells in response to polymyxin B and colistin were in the range of plasma concentrations observed in humans treated with therapeutic doses of the drugs, giving rise to MOEs as low as 0.5 for colistin and 1 for polymyxin B, which would indicate a concern for nephrotoxicity in line with kidney injury observed in patients (Supplementary Table S1). Similarly, MOEs calculated based on *in vitro* PODs obtained from NRK-52E cells and plasma concentrations achieved in rats after exposure to human equivalent doses or doses designed to induce nephrotoxicity were as low as 0.8 for polymyxin and 1.8 for colistin (Supplementary Table S1).

### *In Vitro* Biokinetics of Polymyxin Derivatives

**FIGURE 6:**
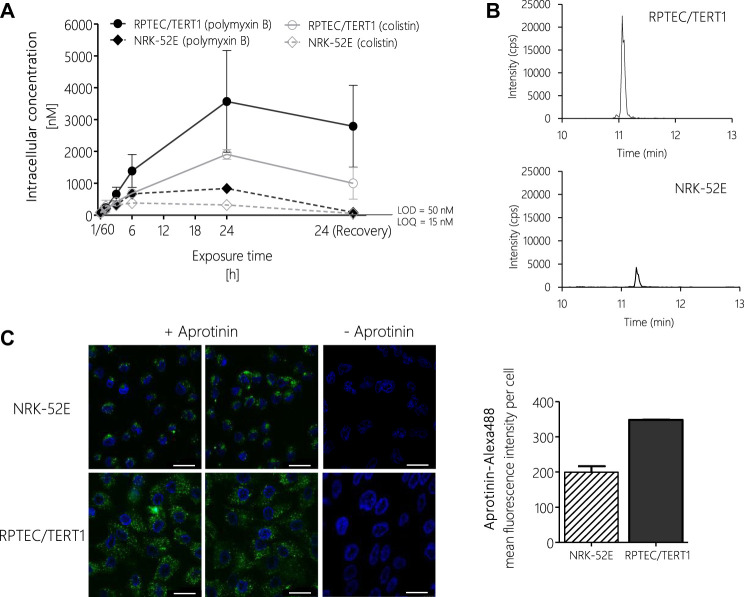
LC-MS/MS analysis of intracellular concentrations of polymyxin B and colistin in RPTEC/TERT1 (―) and NRK-52E (---) cells, showing time-dependent accumulation of polymyxins and significantly higher uptake into RPTEC/TERT1 cells as compared to NRK-52E cells. **(A,B)**. Confocal fluorescence images (scale bar: 25 µm) and fluorescence intensity analysis of Alexa-488 labeled aprotinin uptake (green, left panel) after 4 h incubation of NRK-52E and RPTEC/TERT1 cells with Alexa-488 labeled aprotinin to assess endocytic activity **(C)**, showing increased accumulation of Alexa-488 labeled aprotinin in RPTEC/TERT1 cells as compared to NRK-52E cells. Nuclei were stained with DAPI (blue). Images were acquired with a 63 × 1.4 oil UV objective (scale bar: 25 µm). Data are representative of three independent experiments carried out in triplicates. Data are presented as mean ± SD fold change (*n* = 3).

To test if differences in potency between polymyxin derivatives as well as differences in cytotoxicity in the human vs. the rat cell line were due to differential uptake into proximal tubule cells, intracellular concentrations of polymyxin B and colistin were determined via LC-MS/MS after treatment of RPTEC/TERT1 and NRK-52E cells. In both cell lines, a continuous rise in the intracellular concentration of polymyxin B and colistin was evident within the 24 h treatment period (Figures 6A,B). Compared to the 24 h time-point, the concentrations of polymyxin B and colistin declined during the 24 h off-dose recovery period. Accumulation of polymyxin B and colistin was significantly lower in NRK-52E cells as compared to RPTEC-TERT1 cells (Figures 6A,B), indicating that the differences in cytotoxicity are likely to be related to differences in uptake between the two cell lines. Similarly, in both cell models lower intracellular concentrations of colistin were found as compared to the more toxic polymyxin B.

To understand the basis for the differences in polymyxin uptake between both cell lines, we speculated that the two cell models may differ with regard to the expression of endocytic receptors and/or endocytic activity. Unfortunately, attempts to analyze the mRNA and protein expression of megalin/cubilin via qRT-PCR, immunocytochemistry and Western blot proved to be difficult and provided no conclusive results (data not shown). However, using Alexa-488 labelled aprotinin, which functions as a ligand for the megalin receptor, we were able to demonstrate increased uptake of Alexa-488 labelled aprotinin into RPTEC/TERT1 cells as compared to NRK-52E cells, indicating a higher endocytic activity of the human cell line (Figure 6C).

### Response-Response Analyses to Support *In Vitro* Key Event Relationships and Model Downstream Key Events

**FIGURE 7:**
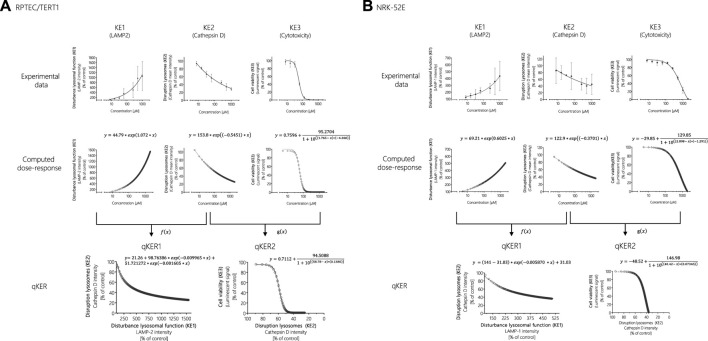
Response-response analysis of polymyxin B KE data obtained in RPTEC/TERT1 **(A)** and NRK-52E **(B)** cells. Based on experimental *in vitro* data on KE1 (LAMP-1/2 intensity), KE2 (cathepsin D intensity), and KE3 (cell viability), additional data points were computed to establish quantitative KER. To this end, KE2 was plotted as a function of KE1 (*f(x)*), while KE3 was plotted as a function of KE2 (*g(x)*).

One way to establish quantitative relationship between the KEs and thus improve quantitative understanding of the causal relationships between KEs is to generate response-response plots (Conolly et al., 2017; Spinu et al., 2020). This allows the relationships to be captured by simple mathematical equations. However, the basic prerequisite for generating response-response plots is an adequate amount of data from the experimental *in vitro* assays collected including application of the same chemical concentrations of test compounds across assays. In order to fulfil these requirements, additional data points were calculated from experimental KE data obtained after polymyxin B treatment in RPTEC/TERT1 and NRK-52E cells (Figure 7). Using the online tool PROAST web (https://proastweb.rivm.nl/), the best-fit function was determined and the regression equations with the corresponding data were displayed in GraphPad Prism 5.01. With the mathematical equations obtained, 400 additional data points in a concentration range between 5 and 2000 µM were computed in 5 µM steps and graphically plotted using GraphPad Prism 5.01 (Figure 7).

These concentration-response curves obtained from polymyxin B data were then used to generate response-response plots for disruption of lysosomes (KE2 - cathepsin D intensity) as a function for the disturbance of lysosomal function (KE1—LAMP-1/2 intensity) (qKER1) and for cell viability (KE3 - cytotoxicity of renal tubular cell) as a function for the disruption of lysosomes (KE2 - cathepsin D intensity) (qKER2) (Figure 7). PROAST web was used to determine the best-fit function and the data and mathematical equation of the response-response curves were then plotted in GraphPad Prism 5.01 (Figure 7).

**FIGURE 8:**
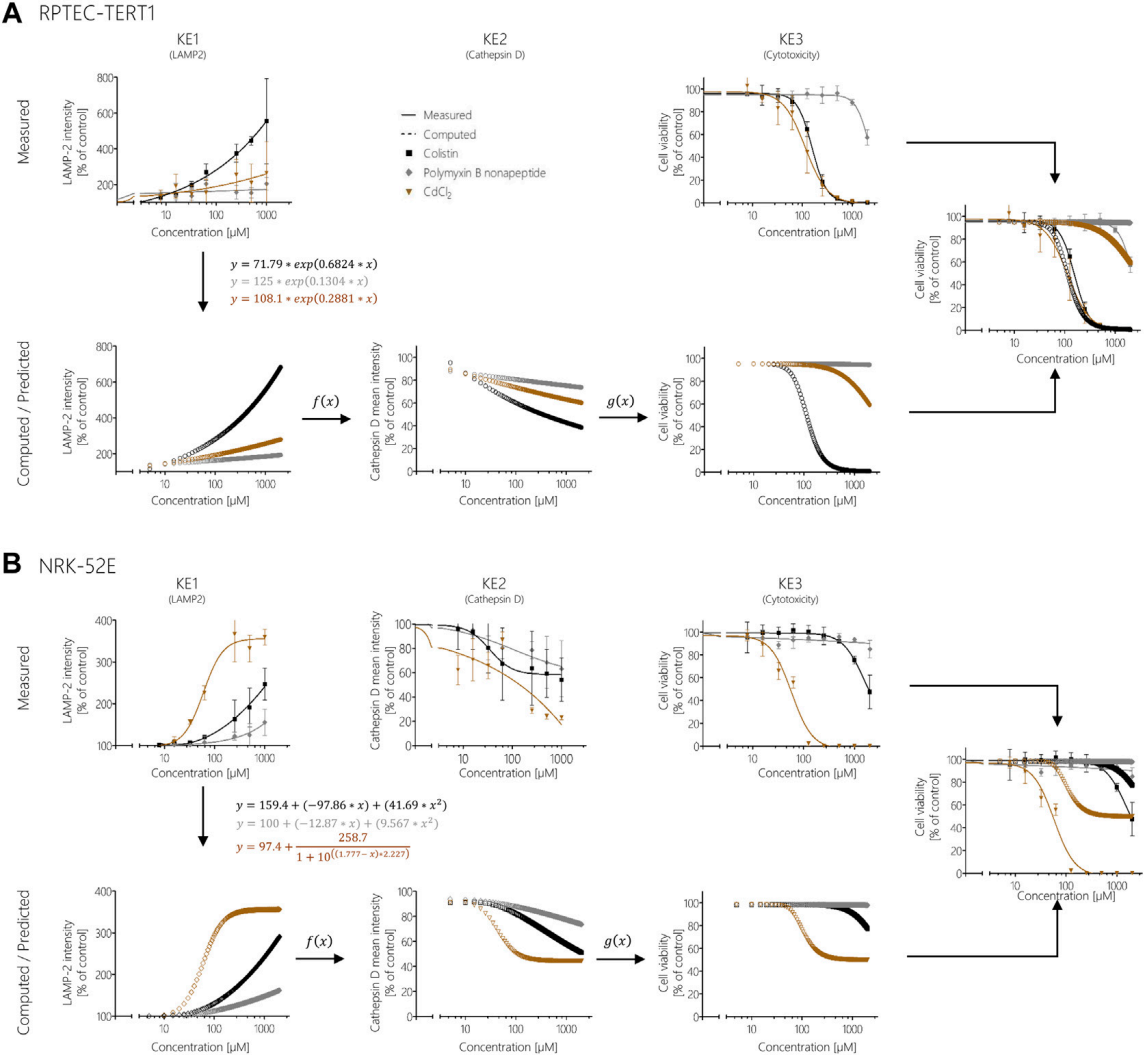
Prediction of colistin, polymyxin B nonapeptide, and CdCl_2_ cytotoxicity using response-response relationships based on polymyxin B data in RPTEC/TERT1 **(A)** and NRK-52E **(B)**. Experimentally determined KE1 data (disturbance of lysosomal function) obtained after treatment of cells with colistin (black), PBNP (grey), and CdCl_2_ (brown) represented by solid lines were used to calculate additional data points (dotted lines) from the obtained mathematical equations for KE1 (disturbance of lysosomal function). Computed KE1 data were then used to predict KE2 (disruption of lysosomes) (dotted lines) using qKER1 (*f(x)*) obtained from polymyxin B data. As a final step, the predicted KE2 data were utilized using qKER2 (*g(x)*) from polymyxin B data to predict KE3 (cytotoxicity) (dotted lines). For better comparison, the dose-response curves of the experimentally determined cytotoxicity (solid lines) and the predicted cytotoxicity (dotted lines) were merged into a single graph.

These KERs were subsequently used to model cytotoxicity (KE3) of colistin, polymyxin B nonapeptide and CdCl_2_ as further potential stressors of the AOP based on experimental KE1 data, i.e., their effects on LAMP-1/2 intensity. This process involved computation of additional data points for experimental KE1 data prior to integration of qKERs to compute the concentration-responses for the downstream KEs (Figure 8). Comparison of the experimentally determined and modelled concentration-response curves for KE3 (cytotoxicity) shows that analysis of disturbance of lysosomal function (KE1) by colistin and polymyxin B nonapetide as an upstream KE and integration of quantitative KERs established from polymyxin B data provided fairly good prediction of colistin and polymyxin B nonapeptide cytotoxicity in both RPTEC/TERT1 (Figure 8A) and NRK-52 cells (Figure 8B), even though there was some concern regarding the cathepsin assay as a reliable marker for KE2. These findings provide empirical support for a causal relationship between the KEs in this AOP.

In contrast, modelling the cytotoxicity of CdCl_2_ based on *in vitro* measurement of KE1 and integration of KER established from polymyxin B *in vitro* data resulted in poor prediction of CdCl_2_ cytotoxicity in both cell lines (Figure 8). In this case, measurement of upstream KE data was found to underestimate CdCl_2_ cytotoxicity.

### Integration of *In Vitro* Biokinetic Data, *In Vitro* Key Event Data and QIVIVE Modelling for Prediction of *In Vivo* Nephrotoxicity of Polymyxin B

In this study, the generic model is applied to simulate human i.v. dose levels of polymyxin B leading to proximal tubule cell concentrations associated with disturbance of lysosomal function (measured by LAMP-2 intensity) and cytotoxicity (measured by CellTiter-Glo®) in RPTEC/TERT1 cells *in vitro*. This exercise is meant as an illustration of how *in vitro* biokinetic data, *in vitro* effect data, AOPs and QIVIVE can be integrated to estimate dose levels associated with acute kidney injury.

**FIGURE 9:**
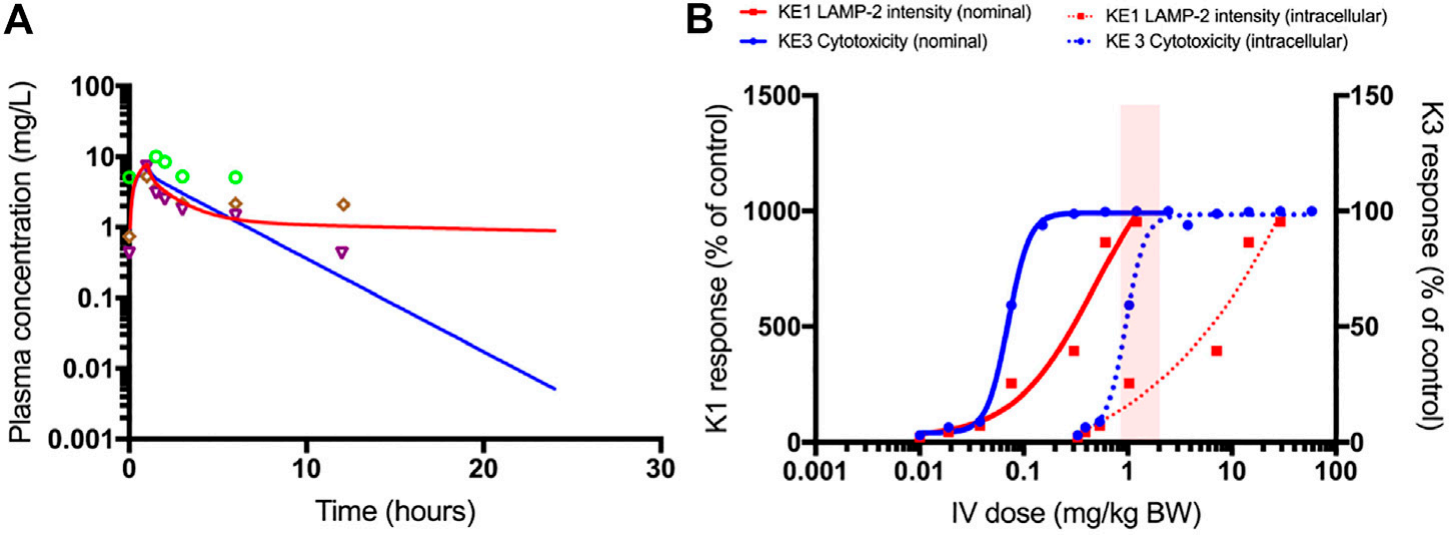
PBPK-model simulated plasma concentrations of 1.0 mg/kg bodyweight polymyxin B, administered by intravenous infusion over 1 h in humans **(A)**. The blue line is the simulation without transporter kinetics (assuming only glomerular filtration). The red line includes active transport to proximal tubule cells. Separate points are measured plasma concentrations obtained from three female patients (Zavascki et al., 2008). QIVIVE based on nominal (solid lines) and cell-associated (dashed lines) polymyxin B *in vitro* effect concentrations **(B)**. The red lines are human bioequivalent dose-response relationships for polymyxin B using results from the *in vitro* LAMP-2 assay in RPTEC/TERT1 cells, the blue lines are human bioequivalent dose-response relationships using results from the *in vitro* CellTiter-Glo^®^ assay with RPTEC/TERT1 cells. The pink bar highlights the clinical authorized dose of polymyxin B, which is associated with nephrotoxic side effects in patients. Note that biological effects on both KEs kick in at similar concentrations, whereas the dose-response concordance between both KE appears to be poorer at higher concentrations at which pronounced cytotoxicity is recorded. This may be due to disruption of lysosomes at these concentrations and time-points. A better concordance between both KEs may be expected when integrating the aspect of time, i.e. assessing LAMP expression at an earlier time-point at which cytotoxicity does not yet occur.

In Figure 9A, the plasma concentration of polymyxin B after 1.0 mg/kg bw *i.v.* administration of polymyxin B in an average human is simulated by the PBPK model. It illustrates the need for accounting for the saturable active reabsorption of polymyxin B suggested to occur via the megalin/cubulin receptor (Nielsen et al., 2016). Renal clearance of polymyxin B is overpredicted when the model only assumes glomerular filtration rate drives excretion of the drug. Ideally, the *in vitro* kidney cell model exhibits comparable transporter activity levels as *in vivo* and can be used to parameterize generic PBPK models with transporter kinetics for secretion and reabsorption. The preservation of expression and activity levels in *in vitro* kidney cell models is rare, and megalin/cubulin expression in RPTEC/TERT1 cells is unlikely to be an exception (Scotcher et al., 2016; Noorlander et al., 2021). This suggests that a scaling of *in vitro* kinetic data from the RPTEC/TERT1 cells with a protein factor and correction for the cortex fraction of the kidney alone does not suffice to predict the excretion-dependent blood concentration of polymyxin B. A relative activity factor (RAF) is therefore needed to correct for potential differences in transporter activity in the RPTEC/TERT1 cells and proximal tubule cells *in vivo*. However, a RAF is not available; there is only evidence for an increased uptake of Alexa-488 labelled aprotinin into RPTEC/TERT1 cells as compared to NRK-52E cells, indicating a higher endocytic activity of the human cell line (Figure 6C). Given the absence of actual data on this scaling factor, the RAF was determined by model fitting to rat and human plasma concentration-time profiles of polymyxin B, illustrating one of the main challenges of accounting for active transport in the kidney in PBPK modelling, the scarcity of reliable human data on scaling factors.

Figure 9B shows the predicted dose-response curves of polymyxin B in patients as well as the *i.v.* dose range administered and associated with nephrotoxicity in the clinic. Human bioequivalent doses were determined using both nominal and cell associated effect concentrations *in vitro*. Simulated human bioequivalent lowest observed toxic doses based on cell-associated concentrations were in line with clinical nephrotoxic dose levels. Human bioequivalent dose levels based on *in vitro* nominal concentrations were more than an order of magnitude lower than reported nephrotoxic doses. This observation may suggest cell-associated concentrations are more representative of biologically effective doses and should thus be used as points of departure (POD) for QIVIVE (Groothuis et al., 2015).

## Discussion

In this work, we used the adverse outcome pathway of kidney injury initiated by receptor-mediated endocytosis and lysosomal overload (Mally and Jarzina, 2022) as a case study to provide a proof-of-concept for the application of the AOP concept to *in vitro* nephrotoxicity assessment. To experimentally support the dose-response and temporal concordance of KEs proposed in this AOP, dose-response relationships were recorded for *in vitro* endpoint selected to cover the KEs after treatment of proximal tubule epithelial cells with polymyxin antibiotics as chemical stressors for this AOP. Concentration-dependent effects were evident across all KEs, with a consistent rank order of biological responses to the three polymyxin analogues. However, the dose-responses obtained from the *in vitro* endpoints were not entirely in concordance, as effects on cathepsin D reflecting KE2 were observed at lower concentrations than those that caused a detectable change in endpoints reflecting KE1. Based on the high biological plausibility of the AOP (Mally and Jarzina, 2022) and methodological issues that may impede accurate analysis of cathepsin D release from lysosomes, we consider that these deviations from dose-concordance may be due to technical reasons rather than wrong biological assumptions. However, this highlights the need to define how well endpoints and assays selected for toxicity assessment actually reflect the KE to be analyzed. In contrast, time-resolved data analysis by high content imaging demonstrated that lysosomal rupture and the decrease in lysosome number occurred prior to the onset of cell death, thus supporting the temporal sequence of key events within this AOP.

Implementation of the AOP conceptual framework for translation of mechanistic data into regulatory decisions requires quantitative understanding of the relationships between KEs within an AOP. We therefore used the developed mechanistic framework and the *in vitro* results obtained from selected KE assays to establish quantitative relationships between KEs in order to test whether modeling of downstream KEs is possible based on an early KE. Since our aim was to provide a proof-of-concept, we focused on cytotoxicity as the most downstream KE in our AOP that can be easily measured *in vitro* and compared to our predictions, although clearly prediction of kidney injury would be the ultimate goal. The quantitative KE relationships established based on polymyxin B data, were used to model downstream KEs for other stressors associated with the same AOP based on experimental KE1 data. The predicted toxicity of colistin in both RPTEC/TERT1 and NRK-52E cells was close to the experimentally determined cytotoxicity. In both cell lines, cytotoxicity of PBNP was low and was also predicted as such, with minor deviations at the highest concentration tested. These exercises demonstrate that prediction of biological responses to structurally closely related compounds that are likely to act by the same mechanism is feasible.

In contrast, prediction of CdCl_2_ toxicity based on early KE data and KER relationships established from polymyxin B KE data significantly underestimated CdCl_2_ cytotoxicity in both cell models. This is perhaps not surprising considering the mechanism of Cd nephrotoxicity. While Cd bound to metallothionein (MT) acts as a ligand for the endocytic receptor and thus shares the same MIE (Wolff et al., 2006), MT is degraded in the lysosomes and Cd is released into the cell, where it can cause functional alterations of a range of proteins due to its high affinity for sulfhydryl groups, leading to disruption of cell signalling and homeostasis (Dorian et al., 1992). Thus, the mechanism of Cd toxicity deviates from this AOP in that disturbance of lysosomal function and lysosomal rupture appear to be only minor contributors to the overall toxic response, in which several AOPs may be at play. This also emphasizes the need for a comprehensive network of AOPs and KE assays for a particular adverse outcome to cover the entire mechanistic landscape of chemically induced kidney injury. Moreover, this example also highlights inherent limitations of *in vitro* test strategies which may not necessarily cover aspects of toxicokinetics that are critical for a particular toxic response, such as binding of Cd to MT required for uptake via receptor-mediated endocytosis.

The importance of *in vitro* biokinetics is further emphasized by the significant differences in biological responses observed between NRK-52E and RPTEC-TERT1 cells treated with polymyxin derivatives, which appear to be due to differential uptake into the cells as evidenced by a lower endocytic activity and lower intracellular concentrations of polymyxin derivatives in the NRK-52E cells as compared to RPTEC-TERT1 cells. Use of nominal concentrations to derive *in vitro* points of departure or as starting points for reverse dosimetry would significantly underestimate the *in vivo* toxicity, whereas reverse dosimetry using intracellular concentration - KE response data as input for deriving human equivalent doses of polymyxin B and colistin provided effective dose levels that are in the range of clinically authorized doses associated with a risk of nephrotoxicity in patients. Overall, this example demonstrates that integration of *in vitro* KE data with *in vitro* biokinetics and quantitative *in vitro* to *in vivo* extrapolation can be a viable approach to human safety assessment. Nevertheless, it will be necessary to define and quantify uncertainties associated with each step of the approach in order to understand where there is a need for refinement or a need for integration of uncertainty factors.

One of the limitations of our work was the lack of incorporation of a time-component, i.e., generation of time-resolved data across all compounds and endpoints, since the time it takes for a change in an upstream KE to trigger a downstream effect is equally important as dose. Response-response relationships were therefore established from KE data obtained at the same time-point, which may not necessarily reflect the time-course of biological responses, such as transient changes in a KE. Such temporal aspects are particularly important when it comes to bridging between KEs measured *in vitro* to KEs and adverse outcome *in vivo*, i.e. in this case the rate of proximal tubule cell death over time will be a critical determinant of the severity of kidney injury. Interestingly, a multiscale quantitative systems pharmacology model was recently reported that may allow prediction of renal dysfuction based on time-resolved urinary biomarker data as a surrogate of proximal tubule cell injury (Gebremichael et al., 2018). Further development of this model may help to bridge the gap between *in vitro* and *in vivo* responses, thereby allowing prediction of *in vivo* nephrotoxicity based on *in vitro* cytotoxicity or upstream KE data obtained in proximal tubule cells.

## Data Availability

The original contributions presented in the study are included in the article/Supplementary Material, further inquiries can be directed to the corresponding author.

## Abbreviations

(q)AOP, (Quantitative) Adverse Outcome Pathway; AO, Adverse Outcome; Col, Colistin; KE, Key Event; LAMP-1/2, Lysosomal Associated Membrane Protein; LMP, Lysosomal membrane permeabilization; MIE, Molecular Initiating Event; PB, Polymyxin B; PBNP, Polymyxin B nonapeptide; POD, point of departure; QIVIVE, Quantitative *in vitro* to *in vivo* Extrapolation.
