# Supplementary material for: Application of the Adverse Outcome Pathway Concept to In Vitro Nephrotoxicity Assessment: Kidney Injury due to Receptor-Mediated Endocytosis and Lysosomal Overload as a Case Study
Source: Front Toxicol. 2022 Apr 19;4:864441. doi: 10.3389/ftox.2022.864441 (PMC9061999; doi:10.3389/ftox.2022.864441)
Supplement: Supplementary file 1 [file DataSheet1.pdf]

Supplementary Material

Table S1 Margin-of-Exposure approach applied to in vitro PODs derived from in vitro KE dose-response data and plasma/serum concentrations (c_{max} or c_{ss}) achieved in humans and animals exposed to therapeutic or known nephrotoxic doses of polymyxin B and colistin.

Treatment	KE	BMC_{10} [µM]	Plasma/Serum Concentration [µM]	MOE
RPTEC/TERT1 / Human				
Polymyxin B	KE1	4.2	0.57 – 4.1 [1,2]	1.0 - 7.4
	KE2	6.8		1.7 - 11.9
	KE3	14.2		3.5 – 24.9
Colistin	KE1	4.3	0.42 – 8.1 [3,4]	0.5 – 10.2
	KE2	-		-
	KE3	23.5		2.9 – 56.0
NRK-52E / Rat				
Polymyxin B	KE1	13.7	7.0 – 7.1 [5]	1.9 – 2.0
	KE2	5.8		0.8
	KE3	19.9		2.8
Colistin	KE1	36.2	5.1 – 10.4 [5,6]	3.5 – 7.1
	KE2	19.1		1.8 – 3.7
	KE3	135.5		13.0 – 26.6

[1] Sandri, A.M., Landersdorfer, C.B., Jacob, J., Boniatti, M.M., Dalarosa, M.G., Falci, D.R., et al. (2013). Population pharmacokinetics of intravenous polymyxin B in critically ill patients: implications for selection of dosage regimens. *Clin Infect Dis* 57(4), 524-531. doi: 10.1093/cid/cit334.

[2] Manchandani, P., Dubrovskaya, Y., Gao, S., and Tam, V.H. (2016). Comparative Pharmacokinetic Profiling of Different Polymyxin B Components. *Antimicrob Agents Chemother* 60(11), 6980-6982. doi: 10.1128/AAC.00702-16.

[3] Tran, T.B., Velkov, T., Nation, R.L., Forrest, A., Tsuji, B.T., Bergen, P.J., et al. (2016). Pharmacokinetics/pharmacodynamics of colistin and polymyxin B: are we there yet? *Int J Antimicrob Agents* 48(6), 592-597. doi: 10.1016/j.ijantimicag.2016.09.010.

[4] Sorli, L., Luque, S., Segura, C., Campillo, N., Montero, M., Esteve, E., et al. (2017). Impact of colistin plasma levels on the clinical outcome of patients with infections caused by extremely drug-resistant Pseudomonas aeruginosa. *BMC Infect Dis* 17(1), 11. doi: 10.1186/s12879-016-2117-7.

[5] Nilsson, A., Goodwin, R.J., Swales, J.G., Gallagher, R., Shankaran, H., Sathe, A., et al. (2015). Investigating nephrotoxicity of polymyxin derivatives by mapping renal distribution using mass spectrometry imaging. *Chem Res Toxicol* 28(9), 1823-1830. doi: 10.1021/acs.chemrestox.5b00262.

[6] Keirstead, N.D., Wagoner, M.P., Bentley, P., Blais, M., Brown, C., Cheatham, L., et al. (2014). Early prediction of polymyxin-induced nephrotoxicity with next-generation urinary kidney injury biomarkers. *Toxicol Sci* 137(2), 278-291. doi: 10.1093/toxsci/kft247.

Fig. S1

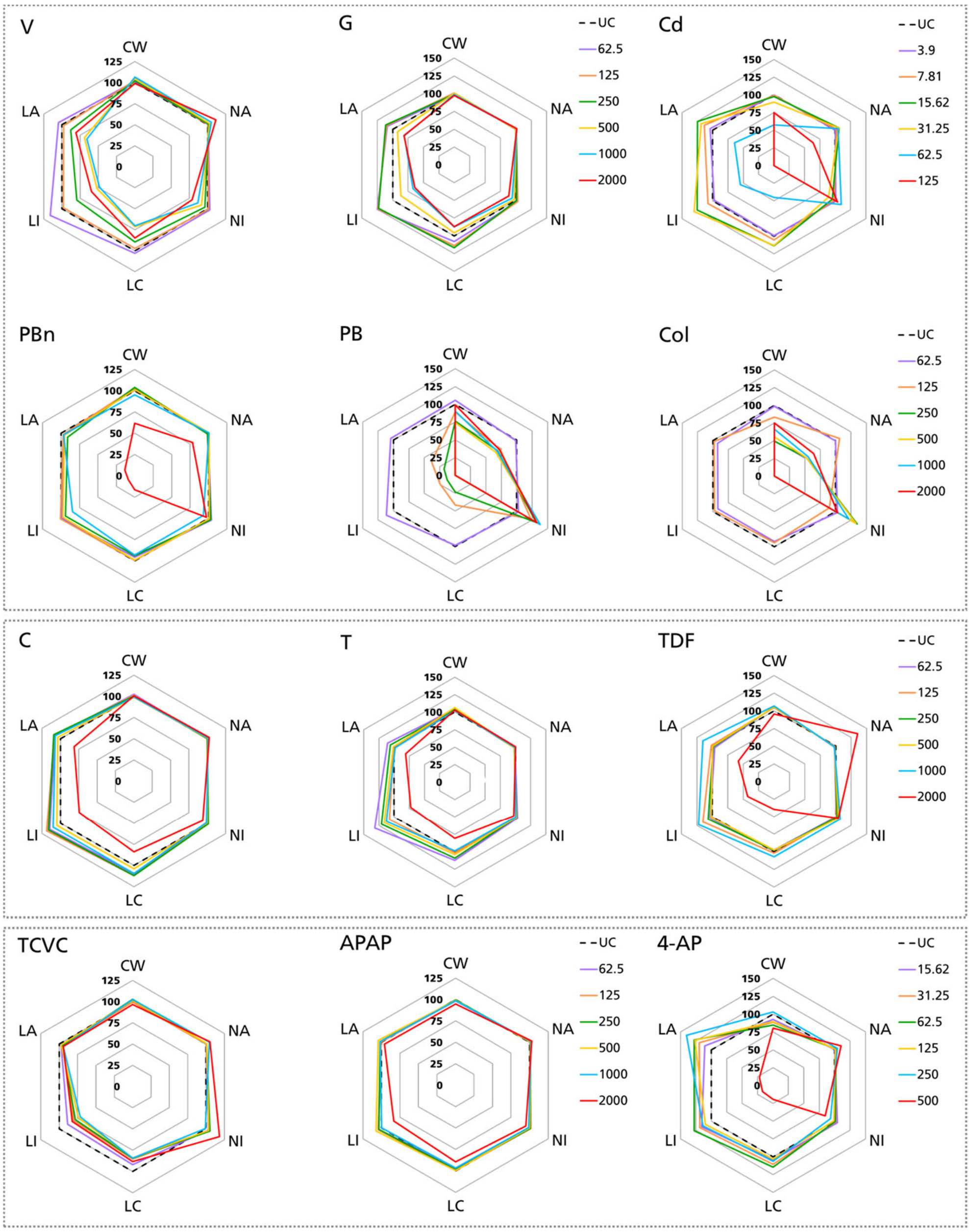

Figure S1 High content screening of the effect of various nephrotoxins on the lysosomal AOP in RPTEC/TERT1 cells. The spider-web diagrams show image analysis data of triplicate experiments for representative concentrations. Cells were treated for 24 hours, except for PB for which the treatment was shortened to six hours due to severe cytotoxicity. All compounds were tested in the range 7.8 to 2000 µM, except for $CdCl_2$ and 4-aminophenol, for which the concentration range tested was smaller due to cytotoxicity. The dotted frames group selected compounds according to reported mode of action: the first two rows show compounds thought to act through the lysosomal AOP, while the other compounds are considered to act primarily via inhibition of mitochondrial DNA polymerase γ (middle frame including C, T, Td) or via covalent protein binding (bottom frame including TCVC, APAP, 4-AP). V = vancomycin; G = gentamicin; Cd = cadmium(II) chloride; PBn = polymyxin B nonapeptide; PB = polymyxin B; Col = colistin; C = cidofovir; T = tenofovir; TDF = tenofovir disoproxil fumarate; TCVC = S-(1,2,2-trichlorovinyl)-L-cysteine; APAP = acetaminophen; 4-AP = 4-aminophenol; UC = untreated control cells; CW = cells per well; NA = nuclei area; NI = nuclei intensity; LC = lysosomes per cell area; LI = lysosome integrated intensity; LA = total lysosome area.
